# A Modified Holder Pasteurization Method for Donor Human Milk: Preliminary Data

**DOI:** 10.3390/nu11051139

**Published:** 2019-05-22

**Authors:** Teresa Capriati, Bianca Maria Goffredo, Marta Argentieri, Liliana De Vivo, Paola Bernaschi, Sara Cairoli, Francesca Laureti, Maria Paola Reposi, Daniela Marino, Sabina Benedetti, Antonella Diamanti

**Affiliations:** 1 Artificial Nutrition Unit, Bambino Gesù Children’s Hospital, IRCCS, Piazza Sant’Onofrio 4, 00165 Rome, Italy; teresa.capriati@opbg.net (T.C.); francesca.laureti@opbg.net (F.L.); mariapaola.reposi@opbg.net (M.P.R.); daniela.marino@opbg.net (D.M.); 2 Biochemistry Laboratory, Department of Specialist Pediatrics, Bambino Gesù Children’s Hospital, IRCCS, Piazza Sant’Onofrio 4, 00165 Rome, Italy; biancamaria.goffredo@opbg.net (B.M.G.); sara.cairoli@opbg.net (S.C.); sabina.benedetti@opbg.net (S.B.); 3 Unit of Microbiology, Bambino Gesù Children’s Hospital, IRCCS, Piazza Sant’Onofrio 4, 00165 Rome, Italy; marta.argentieri@opbg.net (M.A.); paola.bernaschi@opbg.net (P.B.); 4 Clinical Engineering Department, Bambino Gesù Children’s Hospital, IRCCS, Piazza Sant’Onofrio 4, 00165 Rome, Italy; liliana.devivo@opbg.net

**Keywords:** donor human milk, holder pasteurization, human milk bank

## Abstract

Background: Holder pasteurization (HoP) is the recommended method of pasteurization for donor human milk (DHM). The aim of the present study was to compare nutritional and microbiological impact on DHM of a new technique of pasteurization based on technical changes of HoP. Methods: We analyzed milk samples from 25 donors. Each sample, derived from one breast milk expression, was subdivided into three aliquots according to pasteurization: The first was not pasteurized, the second pasteurized by HoP, and the third was pasteurized by modified HoP (MHoP). Each aliquot was assessed as to its microbiological and nutritional profile. Nutritional profile included calcium and triglycerides concentrations detected by spectrophotometry and amino acid levels assessed by high-performance liquid chromatography (HPLC). Results: Triglycerides were significantly lower in pasteurized, by both methods, than in not pasteurized aliquots, while calcium and amino acids concentration were similar. Microbiological profile did not differ between HoP and MHoP aliquots. Conclusions: HoP and MHoP seem to have similar efficacy in preserving some nutritional characteristics of DHM and to confer similar microbiological safety. MHoP is time-saving and potentially costs-effective when compared to HoP, and it is; therefore, potentially of more interest from a practical point of view. Further studies are needed to confirm these findings.

## 1. Introduction

American Academy of Pediatrics (AAP), World Health Organization (WHO), and United Nations Children’s Fund recommend human milk (HM) as the exclusive nutritional source for full-term infants for the first six months and mixed with complementary foods for the first 12 months of postnatal life [1,2,3,4,5,6]. All neonates (including preterm infants) should receive breastfeeding if available; however, donor HM (DHM) is a valuable option if breastfeeding is not available or not sufficient or contraindicated [1,7,8]. Institutional care settings within pediatric wards and intensive care units should pay great attention to the mode of preparation and handling of DHM. Because infants are at high risk to develop infections, due to the incomplete maturation of their immune response [9], DHM is required to be pasteurized to prevent the potential risk diffusion of pathogens [7]. Current guidelines recommend that DHM should be pasteurized by the Holder method (HoP) that allows the maintenance of the temperature above 62.5 °C for 30 min, as required [10,11,12,13,14,15,16,17].

In 2015, in order to shorten the time required for DHM pasteurization and to improve the DHM nutritional profile, we created modifications to HoP by bioengineering changes to the time–temperature curves, one out of the pasteurizer supplied by our Institution, thus obtaining a modified HoP (MHoP) method. The aim of this study was to compare the nutritional and microbiological profiles of DHM samples treated with HoP and MHoP.

## 2. Materials and Methods

### 2.1. Study Design

All women included in the human milk-donating program of our Institution were prospectively enrolled from January 2016 to April 2017. All were mothers of hospitalized babies and were aged from 25 to 37 years. The characteristics of the mothers were according to our Guidelines [17]. A potential donor is considered ineligible to donate milk, according to Italian Ministry of Health Guidelines [17], if she, on the basis of informal interview, clinical evaluation, and specific serological and microbiological testing:

- Currently smokes or uses nicotine replacement therapy;
- Is using, or has recently used, recreational drugs;
- Regularly exceeds recommended alcohol levels for breastfeeding mothers (30–40 mL of spirits, 100 mL of wine, and 200 mL of beer);
- Previously has, or actually is, tested positive for HIV 1 or 2, hepatitis B or C, human T-lymphotropic virus (HTLV) type I or II, or syphilis;
- Has active tuberculosis;
- Six months before donating had unprotected sexual intercourses, blood transfusion, or transplantation or underwent tattoo, piercing, or acupuncture not performed by disposable tools;
- Takes any medication that could represent a risk to the infant’s health;
- Gave a milk sample contaminated.

DHM samples were discarded if they exceeded a count of 10^5^ colony-forming units (CFU)/mL for total viable microorganisms or 10^4^ CFU/mL of *Staphylococcus aureus* or 10^4^ CFU/mL of *Enterobacteriaceae* before pasteurization and any viable microbial count after pasteurization.

**Figure 1:**
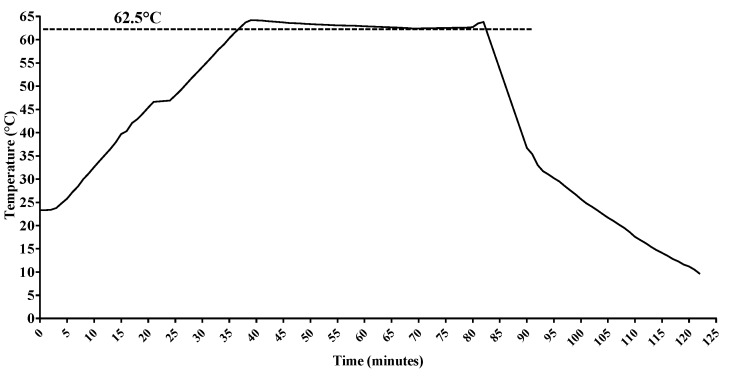
Time–temperature curve for Holder pasteurization method.

**Figure 2:**
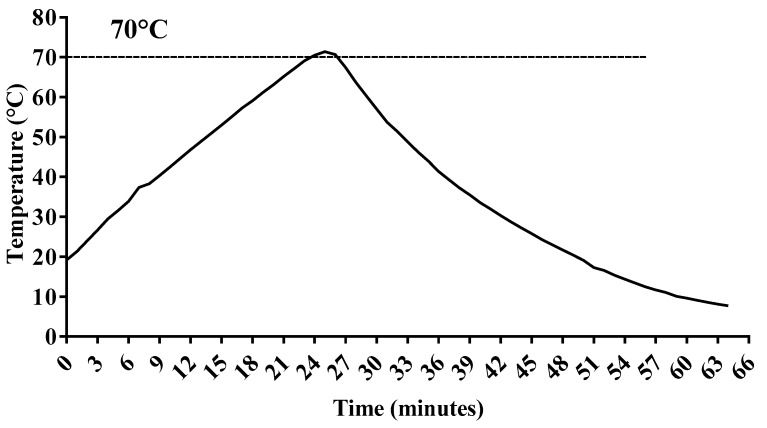
Time–temperature curve for the modified Holder pasteurization method.

DHM samples were prospectively collected. Each DHM sample, derived from one breast milk expression of each donor, was subdivided into 3 aliquots of 30 mL: The first was not pasteurized and stored at −20 °C; the second and the third underwent HoP and MHoP, respectively, and then they were stored at −20 °C before undergoing nutritional evaluations. In Figure 1; Figure 2 we detail the time–temperature profiles according to each method.

Microbiological and nutritional profiles were detected for each aliquot. Nutritional profile included the analysis of calcium, triglycerides, and amino acids (AAs) concentrations.

### 2.2. Nutritional Analysis

Calcium and triglycerides were analyzed, immediately after sampling, by automated assay (ADVIA 2400 Chemistry System, Siemens Healthcare Diagnostics, Forchheim, Germany).

Amino acidic profile was analyzed by reverse-phase high-performance liquid chromatography (HPLC; Agilent Technologies 1200; Waldbronn, Germany). The system consisted of a binary pump, a fluorescence detector, and an auto sampler. A reverse-phase Protocol G123 column (3 µm, P/N H0968, 150 × 4.6 m) was used for the chromatographic separation. The 9-fluorenylmethylchloroformate in acetonitrile (FMOC), o-phthalaldehyde 3-mercaptopropionic acid in borate buffer (OPA) and Borate buffer 0.4 N at pH 10.2 were obtained from Agilent. For the separation, a mobile phase A (12 mM Na2HPO4 in 1 L water, added to 1.8 mL THF, at pH 7.23) and a mobile phase B (12 mM Na2HPO4 in 500 mL water at pH 7.23 added to 350 mL methanol and 150 mL acetonitrile) in gradient were used. A mixture of acidic, neutral, and basic amino acids standard was used as calibrator for the separation.

For the sample preparation for reverse phase chromatography, 500 µL of sample was deproteinized by ultrafiltration, using Amicon Ultra Centrifugel Filters Ultracel-3K, at 13000 rpm for 9 min. A total of 60 µL of filtrate was added to 60 µL of Internal Standard (Norvaline 0.1 mM) and 60 µL of Borate Buffer. A total of 180 µL of the mixture was transferred into vials and placed into the auto sampler of the HPLC system.

After the derivatization, 4.8 µL of the mixture was injected for each chromatographic separation. Amino acids were derived with OPA and FMOC and detected by fluorescence detector with excitation = 340 nm and emission = 450 nm. The chromatographic separation was obtained using a gradient of dilution and a column temperature of 40 °C. The flow rate was kept at 1.0 mL/min throughout the analysis, the run time was 41 min.

### 2.3. Microbiological Analysis

Firstly, milk samples were inoculated into Columbia agar + 5% sheep blood, MacConkey agar, Chocolate agar + polyvitex, and Tryptone soya agar, and then they were incubated at 37 °C for 48 h. Microbial count was detected for each subculture. Bacteria were identified with technology matrix-assisted laser desorption ionization-time of flight mass spectrometry (MALDI-TOF MS), with Microflex LT (Bruker Daltonik GmbH, Bremen, Germany). Positive cultures were expressed as quantitative colony count from 10^3^ to >10^5^ CFU/mL.

### 2.4. Ethics

All donors were mothers of hospitalized babies, thus they gave, at admission, their written consent to do not interventional studies in line with the rules of our Institution that is authorized by the Ministry for Health Care for research and clinical studies. This procedure satisfies the indications from the “Bambino Gesù” Hospital Ethical Committee.

### 2.5. Statistical Analysis

All data are presented as mean ± SD. ANOVA test was applied to evaluate differences in AAs, calcium, and triglycerides between the three groups of aliquots (not pasteurized, pasteurized by HoP, and pasteurized by MHoP). In presence of differences suggested by the preliminary ANOVA, we applied a nonparametric test to complete the evaluation, because we considered that our data were distributed by a non-Gaussian way. Thus, the Mann–Whitney test was used to detect differences in calcium, triglycerides, and amino acids levels between specific groups. *p* value of <0.05 was considered statistically significant. Statistical evaluation and figures were performed using Graph Pad 6 for Windows.

## 3. Results

Overall, 81 milk aliquots (27 not pasteurized, 27 pasteurized by HoP, and 27 pasteurized by pasteurized by MHoP) derived from 27 breast milk samples of 25 donors were analyzed during the study-period. Two donors provided two milk samples (see below) and; therefore, the overall milk aliquots available for the analysis were 81. Seventy-five aliquots (25 not pasteurized, 25 pasteurized by HoP, and 25 pasteurized by MHoP) derived from 25 donors were assessed for nutritional analysis; microbiological analysis was available for 56 milk aliquots (19 not pasteurized, 18 pasteurized by HoP, and 19 pasteurized by MHoP) derived from 19 donors (for one donor the pasteurized by HoP aliquot was not available). Two women were considered not eligible to donate because the not pasteurized aliquot results showed colonization by >10^5^ CFUs/mL of *Staphylococcus aureus* in the first and by *Bacillus cereus,* persisting after HoP and MHoP, in the second one. Both gave a second milk sample after two weeks and the not pasteurized aliquot from the first woman resulted as clean, thus she was readmitted to the donating program. The sample from the second woman resulted persistently contaminated by *Bacillus cereus* before pasteurization and after HoP and MHoP, thus this woman was definitively excluded from the donating program.

### 3.1. Nutritional Profile

**Figure 3:**
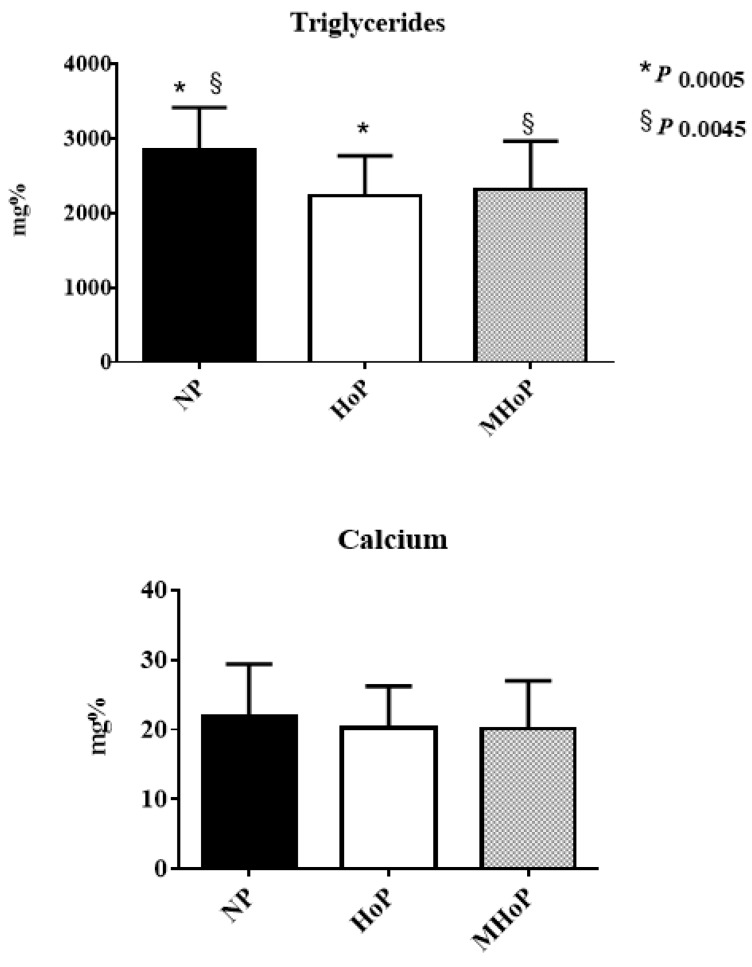
Triglycerides and calcium concentration in not pasteurized, pasteurized by Holder method, and pasteurized by modified Holder method aliquots. NP—not pasteurized; HoP—Holder pasteurization; MHoP—modified Holder pasteurization.

**Figure 4:**
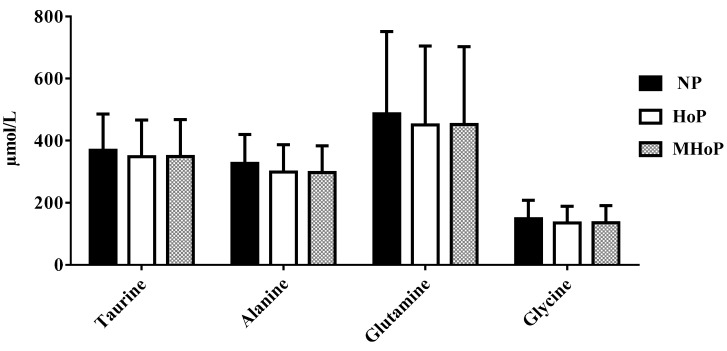
Amino acids concentration in not pasteurized, pasteurized by Holder method, and pasteurized by modified Holder method aliquots. NP—not pasteurized; HoP—Holder pasteurization; MHoP—modified Holder pasteurization.

**Figure 5:**
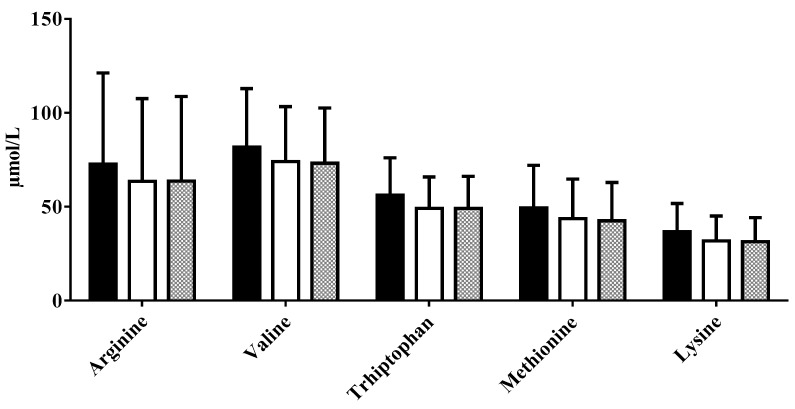
Amino acids concentration in not pasteurized, pasteurized by Holder method, and pasteurized by modified Holder method aliquots. NP—not pasteurized; HoP—Holder pasteurization; MHoP—modified Holder pasteurization.

**Figure 6:**
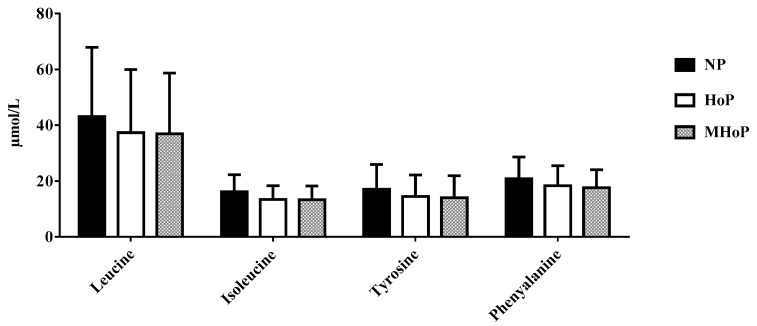
Amino acids concentration in not pasteurized, pasteurized by Holder method, and pasteurized by modified Holder method aliquots. NP—not pasteurized; HoP—Holder pasteurization; MHoP—modified Holder pasteurization.

Calcium and AA concentrations were similar in the three aliquots (see Figure 3, Figure 4, Figure 5 and Figure 6 see below). Only triglyceride content resulted significantly lower in pasteurized aliquots when compared to the not pasteurized ones (2836 ± 5799.0 mg%). Aliquots MHoP treated, nevertheless, maintained higher levels of triglycerides (2317 ± 649.2 mg%) than aliquots HoP treated (2238 ± 530.4 mg%). 

### 3.2. Microbiological Profile

**Figure 7:**
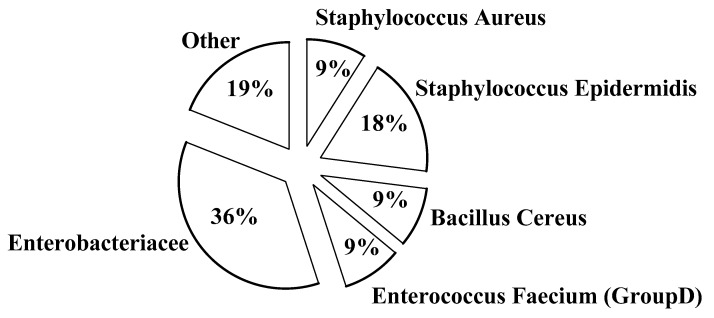
Bacterial species and count. CFUs–colony forming units/mL of donor human milk.

Eight out of the 25 donors (32%) gave contaminated milk samples before pasteurization and two women were excluded from the donating program (see above). In Figure 7 we detail the spectrum of bacterial species in not pasteurized aliquots. However, all the samples proved sterile following pasteurization by both methods. Only *Bacillus cereus* persisted after HoP and MHoP treatment.

## 4. Discussion

For the safe feeding of fragile infants, such as preterm infants, great attention should be paid to the modes of preparation and handling of foods. In particular, DHM requires careful management to prevent the risk of nutritional inadequacy and infections [9]. In this study we assessed the impact of a new method of pasteurization derived from the classical HoP on some of the nutritional characteristics and on the microbiological profile of DHM. Our preliminary results show that HoP and MHoP confer similar nutritional adequacy and microbiological safety to DHM, although MHoP seems to save more triglycerides than HoP. 

With regards to the nutritional profile, we focused on AA and fat concentration because carbohydrates seem to not be affected by pasteurization [18]. In view of its great relevance for the bone maturation of preterm infants [19], we identified calcium as a relevant marker of the nutritional safety of this new method. 

Dietary lipids are the main source of energy in infants with a total of 45–55% of the total energy provided by human milk and formula in the first six months of life. They are involved in the regulation of cell functions, in inter- and intracellular communication, and in the epigenetic modulation of the genome [20]. The anatomical and functional development of the nervous system seems to depend on the direct supply of lipids [20]. For all these reasons, triglycerides concentration was included in the first step of the evaluation of this new method. Conflicting results are reported about the residual fat content in human milk after pasteurization [21,22,23,24,25,26,27,28,29,30,31]. Our study found 18.3% and 21% of triglycerides were lost after HoP and MHoP, respectively, suggesting that MHoP may be advantageous in terms of fat saving. 

So far one only study, according to our findings, reported calcium concentration unchanged after pasteurization [32].

Furthermore, several reports overall found that pasteurization does not impact on the AA profile, in line with our results [21,24,29,33,34,35]. Some reports found little changes in glutamine, arginine leucine, aspartate, and lysine concentration after HoP [24,34,35].

From a microbiological point of view, we found that more than 30% of all donors gave contaminated milk samples, that, nevertheless, were completely cleaned by both the pasteurization methods, except for *Bacillus cereus*. According to our National guidelines, we excluded from the donating program the two women who gave samples contaminated by *Bacillus cereus,* and by more than 10^5^ CFUs/mL of *Staphylococcus aureus* [17]. However, the pasteurization completely cleaned *Staphylococcus aureus* but not *Bacillus cereus.* This finding may suggest the need of a re-modulation of the exclusion criteria for the human milk donating programs and in particular that the contamination by *Bacillus cereus* should be a pre-requisite of exclusion. The rate of contamination before pasteurization found in the present survey is similar to that reported in previous studies, which identified generally coagulase-negative *Staphylococcus* and Gram-negative rods in human milk samples [9,36] (see Table 1 for details).

When screening is performed before pasteurization, milk banks may opt to discard any raw milk that contains organisms or potential pathogens that can produce heat-stable enterotoxins, endotoxins, and spores [37]. The practice of obtaining cultures before pasteurization is not followed consistently in milk banks globally for simple cost-benefit reasons. Milk banks may forego the pre-pasteurization testing to save time and money and to preserve a larger supply of raw milk. Milk banks that test only after pasteurization may yield more product because milk is not discarded un-necessarily before pasteurization. Using the practice of culturing milk before and after pasteurization in a study conducted in France, Dewitte and colleagues [38] reported positive after pasteurization bacterial growth rates of 0.5%. However, 10.8% of DHM in milk banks in France is discarded before pasteurization after initial bacteriologic screening [38]. Similarly, the Taipei City Hospital Milk Bank in Taiwan reported that 0.63% of DHM had positive test results after pasteurization. At that facility, 27.9% of DHM was discarded after pre-pasteurization screening [39]. The Perron Rotary Express Milk Bank (King Edward Memorial Hospital, Perth, Australia) reported post-pasteurization bacterial growth rates of 2.4%; 26.4% of raw DHM was discarded before pasteurization, and only 0.9% of the discarded DHM contained *Bacillus* species [37]. Landers and Updegrove [36] reported a post-pasteurization bacterial growth rate of 7%, and *Bacillus* species was the predominant contaminant (5%) in DHM from the Austin Mothers’ Milk Bank (Austin, Texas). Among their total sample of 17 batches of donor milk, 10 batches of donor milk had positive test results for Bacillus species before and after pasteurization, and the remaining seven had positive test results only after pasteurization [36]. Finally, Jang et al. [40] cultured samples after pasteurization at the Gangdong Kyung Hee University Milk Bank (Seoul, Korea); they reported a bacterial growth rate of 12.6% and cataloged the majority as *Bacillus* species. These findings indicate low rates of positive results for post pasteurization milk cultures; however, a large volume of milk in each of the reports was discarded before pasteurization, which indicates a potential waste of milk.

In the present experience, we modified one HoP pasteurizer by changing the time–temperature curve to reach temperatures close to 72.5 °C. HoP is not the only method of pasteurization of DHM and new methods have been investigated that try to improve the biological quality and safety of DHM [41,42]. Alternative techniques are based on thermal methods such us high-temperature short-term (HTST) pasteurization, that is flash pasteurization to 72 °C for 5–15 s [33,35,43,44,45], and its homemade low-tech variant for developing countries (flash-heat treatment) [46,47,48] and not thermal methods such us ultrasonic processing [49]. HTST pasteurization is only employed for industrial preparations but not in clinical settings. A recent Italian experience reports, nevertheless, the use of this industrial method optimized for the use in clinical settings [50]. High-pressure processing (HPP) [51,52], ultraviolet irradiation (UV) [53,54,55], and Ohmic heat treatment [41] are methods that have also been investigated.

The main limitation of our study is that the nutritional analysis was restricted to the detection of amino acids, calcium, and triglycerides, while it would be of interest to compare caloric density, fat soluble vitamins, and carotenoid levels between methods of pasteurization. This analysis will be part of a second step of evaluation of MHoP.

In conclusion, DHM, essential for the nutrition of sick and preterm babies, requires procedures, historically represented by the HoP method, that warrant microbiological safety. MHoP seems to have the same microbiological safety and seems to determine similar nutritional changes than HoP. However, MHoP has the advantage of being less time-consuming and probably more cost-effective than HoP. Therefore, it could be very valuable in Institutions where a great number of milk preparations are required daily. The shorter length of the MHoP process is probably responsible for the lower amount of fat lost. Other nutritional aspects should be evaluated in the future to confirm our preliminary results.

## Figures and Tables

**Table 1:** Practice and rate of contamination of donor human milk.

	Contamination Rate BP (%)	Contamination Rate AP (%)	Bacillus Species Rate AP (%)	DHM Discarded Rate (%)
Perth, Australia (Hartmann et al. 2017)	26.4	0.9	NR	27.3
Seoul, Korea (Jang et al., 2016)	NR	12.6	Several cases	12.6
Nord-Pas-de-Calais France (Dewitte et al., 2015)	10.8	0.5	NR	11.3
Taipei, Taiwan (Chang et al. 2013)	27.9	0.63	NR	28.5
Children’s Hospital of Philadelphia	NR	5.8	5.8	5.8
Austin, Texas, United States (Landers and Updegrove, 2010)	NA (pooled)	7	5	7

BP—before pasteurization; AP—after pasteurization; DHM—donor human milk; NA—not applicable; NR—not reported.
